# Editorial

**Published:** 1846-04

**Authors:** 


### THE MEDICAL EXAMINER.

PHILADELPHIA, APRIL, 1846.

---

### TO CORRESPONDENTS AND PUBLISHERS.

Several communications and notices of new books, intended for the present number, are unavoidably postponed, but will appear in the next. So arduous have been the editor's labours of late that it has been out of his power to pay his wonted attention to this department of the journal; and he craves a little indulgence, and promises better for the future.

---

### HEALTH OF PHILADELPHIA.

We have thought it proper to speak particularly of the health of the city of late, in consequence of the exaggerated reports that have been spread throughout the country of the prevalence of small-pox. That disease has so nearly disappeared, that it can no longer be regarded as epidemic; and in other respects, the inhabitants were never more exempt from sickness than at present.

---

### NATIONAL MEDICAL CONVENTION.

In another part of the present number of the Examiner, we have inserted, by request of the Chairman of the committee of the N. Y. State Medical Society, the *Report* submitted to that body, at its recent meeting, by the committee appointed to carry out its resolution passed last year, in relation to a National Medical Convention to be held in the city of New York, in May next. The committee state that "The Medical Schools of Philadelphia are the only ones from whom replies have been received, that decline sending delegates, and giving a hearty support to the proposed measure." How many "Medical Schools" there are elsewhere from which no replies have been received, and that are therefore not likely to send delegates, the committee has not told us; but if our information is correct on the subject, the number of schools and societies who have either declined or not replied, is so great as to render it doubtful whether the Convention that shall assemble will be so composed as to entitle it to be regarded as *National* in its character. "The wish is *not* father to the thought"—on the contrary, we should rejoice if a properly organized Convention, representing all the interests of the profession in the United States, could be assembled, and with pure motives and calm deliberation, devise some scheme for improving the condition of the profession throughout the whole country. Such a Convention would indeed clothe itself with honour, and confer lasting benefits upon the profession and the people. But to accomplish the objects for which alone it should be constituted, the Convention must be held under circumstances calculated to excite no jealousies or distrust—must be held in a central place, if not in the capital of the nation—and must be utterly disconnected with all *local interests* and *personal aggrandizement.*

While the Medical Schools of Philadelphia, for reasons which to them have seemed sufficient, have declined taking part in the Convention now proposed to be held, if that body shall devise any good and practical scheme for bettering the condition of the profession, they will not be the last to adopt it, or the least faithful in carrying it out. As things are, however, they are uncommitted, and will judge impartially of the doings of the New York Convention, when they are before them.

